# Artificial intelligence–powered three-dimensional echocardiography in clinical practice: the illusion of automation?

**DOI:** 10.1093/ehjimp/qyag123

**Published:** 2026-07-07

**Authors:** Andrea Barbieri, Francesca Mantovani, Federico Fortuni

**Affiliations:** Cardiology Division, Department of Biomedical, Metabolic and Neural Sciences, University of Modena and Reggio Emilia, Policlinico di Modena, Modena 41125, Italy; Cardiology Unit, Azienda USL-IRCCS di Reggio Emilia, Viale Risorgimento 80, Reggio Emilia 42123, Italy; Cardiology and Cardiovascular Pathophysiology, S. Maria Della Misericordia Hospital, University of Perugia, Perugia 06121, Italy

Three-dimensional echocardiography (3DE) for chamber quantification has evolved from a promising technique to a practical quantitative tool, particularly through artificial intelligence (AI)–based automated analysis.^1,2^ These systems provide rapid, reproducible chamber measurements without geometric assumptions, addressing the limitations of two-dimensional echocardiography.^3^ Yet, a paradox remains: although AI-powered 3DE may transform cardiac morphometry,^4^ implementation remains inconsistent.^5^ Based on extensive real-world experience with the routine implementation of AI-assisted 3DE analyses at our facility, we argue that insufficient standardization of 3DE acquisition and analysis remains a major barrier to the reliable clinical implementation of AI. Indeed, in this field as well, clinical practice often differs substantially from theory. To understand this gap, we should return to the basics: even the most complex AI-driven and automated analyses ultimately depend on the quality of the raw data entered into the system. Therefore, how images are acquired, selected, and processed remains crucial, as automation cannot compensate for suboptimal or non-standardized input data.^6^ This limitation can be overcome only through structured education and rigorous standardization, particularly in the acquisition of both 2D and 3D echocardiographic images. High-level expertise and dedicated training are essential for effective 3DE implementation in clinical practice and represent the prerequisite for reliable automated AI-driven analysis. Without appropriate education, standardized acquisition protocols, and expert quality control, automation risks amplifying variability rather than reducing it.

Automated AI-3DE measurements are highly reproducible. However, reproducibility does not necessarily translate into reliability in routine practice, where acquisition, dataset selection, operator interaction, and test–retest variability reintroduce uncertainty.^7^ Not all parameters perform equally. Left ventricular (LV) volumes generally show the best accuracy and reproducibility, whereas ejection fraction (EF) is more vulnerable to volumetric errors, and left atrial (LA) volumes remain the least robust measurements. Overall, robustness follows a clear hierarchy: LV volumes > EF > LA volumes.^7^ Automated AI-3DE performance depends on the input domain,^8^ namely, the spectrum of image acquisition conditions, scanner settings, vendors, image quality, acoustic windows, cardiac rhythms, ventricular geometries, and patient characteristics represented during algorithm development and validation. Because most models are trained on specific data distributions, their performance may deteriorate when applied to images or populations that differ from those used for training, a problem known as domain mismatch.^9,10^ In clinical practice, this mismatch may arise from subtle variations in acquisition protocols, operator experience, temporal or spatial resolution, chamber foreshortening, gain settings, stitching artefacts, or disease phenotypes that were under-represented in the original dataset. Importantly, such degradation may not be immediately apparent to the user: the software may still generate precise-looking contours, volumes, and EFs, thereby preserving the appearance of automation while progressively losing accuracy. This phenomenon, often referred to as silent drift, is particularly concerning because it can occur gradually as equipment, software versions, acquisition habits, and patient populations change over time. Thus, automation does not remove the need for expertise; rather, it shifts expertise upstream, towards standardized acquisition, careful image selection, continuous quality control, and periodic validation against reliable reference standards.

Image quality is the primary determinant of AI performance in 3DE.^2,3^ It depends on endocardial delineation, dataset completeness, signal-to-noise ratio, and the absence of dropout, not on volume rate alone. However, image quality is still largely assessed visually and semi-quantitatively rather than by objective metrics. Consequently, AI reduces measurement variability but remains dependent on operator-driven image selection. Changes in scanners, transducers, or software may alter image characteristics and create a device-driven domain shift, even when the same patient is analysed. This device-driven domain shift means that AI performance is not a fixed property of the algorithm but an emergent property of the imaging ecosystem. New transducers or platforms should be followed by validation, recalibration, and monitoring of measurement stability.^11^ Phenotype-specific domain mismatch may also occur and is particularly relevant when automated measurements are used to guide treatment decisions. Hypertrophic cardiomyopathy, and especially hypertrophic obstructive cardiomyopathy, represents a paradigmatic example because its ventricular geometry, asymmetric hypertrophy, small cavity size, hyperdynamic systolic function, and frequent endocardial-tracking challenges differ substantially from the populations typically included in general AI-3DE training datasets. In this setting, automated LV EF estimates may be systematically biased rather than merely imprecise, and this becomes clinically critical when measurements are close to predefined therapeutic thresholds. This issue is especially important in the context of disease-modifying therapies such as cardiac myosin inhibitors, including Mavacamten, whose initiation, dose adjustment, interruption, or discontinuation may depend on protocol-defined LV EF cut-offs.^12,13^ If an AI-derived EF value is inaccurate or biased in a patient with hypertrophic cardiomyopathy, the algorithm may directly influence decisions to continue, reduce, or stop therapy. Because the algorithms used to derive EF have not necessarily been specifically validated in HCM populations, their uncritical implementation may have deleterious consequences, including inappropriate treatment interruption or unsafe continuation of therapy. Thus, the key limitation is not only random measurement error, but systematic bias around clinically meaningful decision thresholds. Since EF is inherently method-dependent and influenced by image acquisition, contour definition, ventricular geometry, and software assumptions, AI may convert small systematic errors into reproducible but misleading measurements. Therefore, validation must be phenotype-specific and use-case-driven, particularly when automated outputs are used for regulatory, therapeutic, or safety decisions.

A pragmatic framework for AI-3DE should stratify implementation by operator expertise, image quality, phenotype, and technological context (*Figure 1*). Inexperienced operators should not use AI alone, and novices require expert supervision. When image quality is suboptimal, manual measurements remain preferable. High-quality datasets can support automated quantification, but real-world practice often falls short of this standard. As operators gain confidence, they may accept lower-quality datasets and rely more on manual corrections, increasing feasibility while reducing reproducibility. Objective quality control tools are needed to automatically identify and exclude datasets with insufficient image quality, thereby preventing unreliable analyses. Reliable implementation also requires harmonized protocols, thresholds for manual intervention, cross-vendor validation, structured training, and continuous quality control.

**Figure 1:**
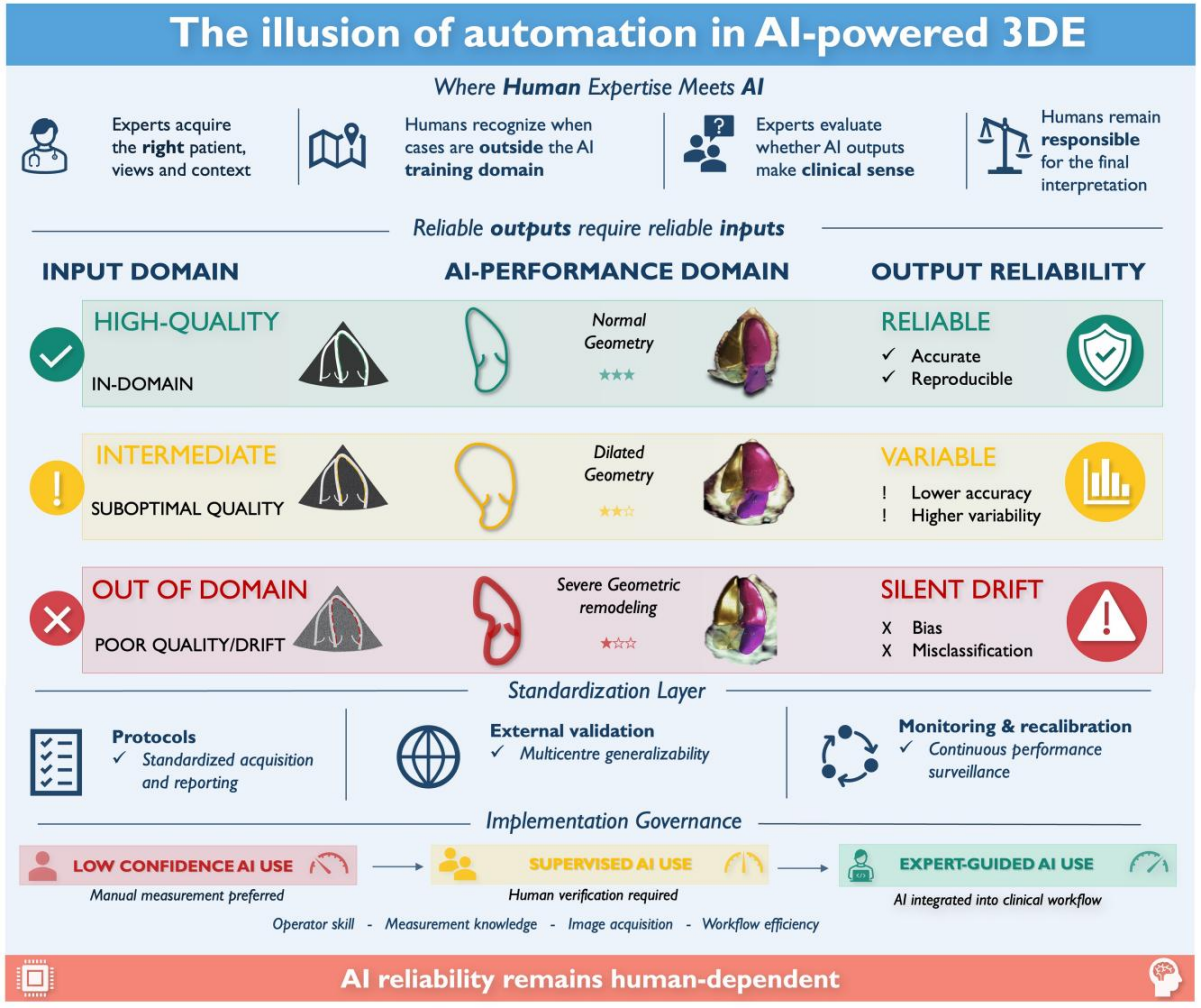
The Figure shows that AI output reliability depends on input image quality and anatomical suitability: high-quality inputs produce reliable results, intermediate-quality inputs yield variable accuracy, and poor-quality or out-of-domain inputs may result in "silent drift" and misclassification. The figure emphasizes the need for standardized acquisition, external validation, continuous monitoring, and expert human oversight, concluding that AI reliability remains human-dependent.

In conclusion, AI-3DE should be implemented as an expert-supervised tool, not as a shortcut for inexperienced use. Its routine application should require standardized 2D and 3D acquisition, adequate operator training, visual verification of automated contours, and objective image quality control. Software should also provide reliability warnings when analyses are performed outside the validated input domain and should reject datasets with inadequate image quality. These safeguards are essential to prevent silent drift and ensure that automation improves, rather than compromises, clinical decision-making.

## Data Availability

No new data were generated or analysed in support of this research.
